# Near-Infrared Spectroscopy in Food Analysis: Applications, Chemometric Strategies, and Technological Advances

**DOI:** 10.3390/foods15101814

**Published:** 2026-05-20

**Authors:** Limin Dai, Dong Luo, Jun Zhang, Yuan Chen, Changwei Li

**Affiliations:** 1 School of Agricultural Engineering, Jiangsu University, Zhenjiang 212013, China; lmdai@ujs.edu.cn (L.D.); 13082872915@163.com (D.L.);; 2 School of Mechanical and Electrical Engineering, Jiaxing Nanhu University, Jiaxing 314001, China; 11613006@zju.edu.cn

**Keywords:** near-infrared spectroscopy, chemometric analysis, food quality assessment, non-destructive detection, fuzzy clustering

## Abstract

This paper presents a comprehensive review on near-infrared (NIR) spectroscopy applied in food analysis, systematically elaborating its core principles, widespread industrial applications, advanced chemometric strategies, and cutting-edge technological progress. NIR spectroscopy (760–2500 nm), characterized by rapid, non-destructive detection and minimal sample preparation, has been widely implemented in quality evaluation and safety monitoring of grains, meat, fruits and vegetables, dairy, fermented products, tea, coffee, and other processed foods, realizing quantitative analysis of nutrients, freshness assessment, texture prediction, adulteration identification, origin tracing, and rapid preliminary screening of toxin/pesticide residues. A series of chemometric methods, including spectral preprocessing (SNV, MSC, S-G smoothing), feature extraction, and variable selection (CARS, PSO-CMW, ICPA), as well as linear/nonlinear modeling algorithms (PLS, SVM, BP-ANN, fuzzy clustering) significantly boost the accuracy and robustness of spectral analysis. Meanwhile, portable NIR devices and online monitoring systems promote on-site and real-time detection in food supply chains. Despite existing challenges such as calibration transfer, matrix interference, and model generalization, innovations like multimodal data fusion, deep learning integration, and intelligent algorithm optimization offer effective solutions. This review not only summarizes the latest research advances of NIR technology in the food field but also emphasizes its significant advantages as a rapid, non-destructive complementary tool to traditional destructive detection methods, providing theoretical support and technical reference for accelerating the industrial translation and standardized application of NIR spectroscopy, and ultimately safeguarding global food quality and safety.

## 1. Introduction

### 1.1. Significance of Food Quality and Safety Monitoring

Ensuring food quality and safety is paramount in modern food systems, as consumer demand for traceable, nutritious, and unadulterated products continues to grow [1,2]. Contaminants such as mycotoxins [3], microbial spoilage in meat [4], and adulteration in processed foods [5] pose significant health risks and economic losses. Traditional analytical methods such as high-performance liquid chromatography (HPLC) and enzyme-linked immunosorbent assay (ELISA) are accurate but limited by tedious sample preparation, high cost, and destructive testing, which restricts real-time monitoring in the food supply chain [6,7]. Therefore, rapid, non-destructive, and cost-effective techniques are urgently needed to overcome the above limitations and realize real-time monitoring in the food supply chain [8]. Near-infrared (NIR) spectroscopy technology, with its unique advantages of being fast, non-destructive, and efficient, has gradually developed into a research and application hotspot in the field of food quality testing [9,10].

### 1.2. Evolution and Advantages of NIR Spectroscopy in Food Science

**Figure 1:**
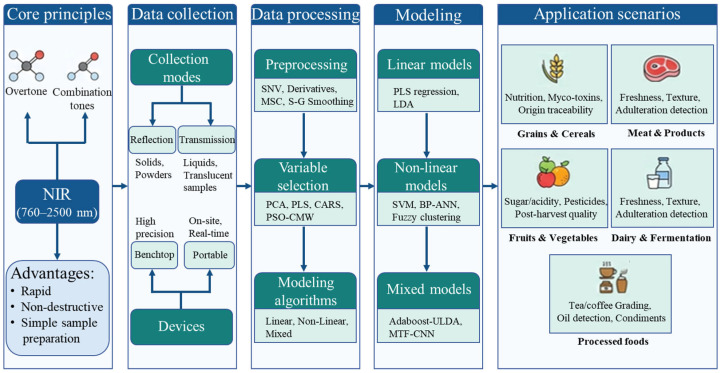
Core principles, data processing workflows, and typical application scenarios of NIR spectroscopy in food analysis.

NIR spectroscopy has emerged as a transformative tool in food analysis, leveraging the absorption of NIR radiation (760–2500 nm) by functional groups in organic compounds [11,12]. This technique offers distinct advantages, including non-destructive measurement, minimal sample preparation, high throughput, and portability, enabling online and in-line analysis [13]. Early applications were limited to quantitative analysis of major components (e.g., moisture and protein in grains), whereas recent advances in instruments and chemometrics have extended its scope to complex tasks such as adulteration detection, variety classification, and freshness evaluation. For instance, NIR coupled with fuzzy discriminant analysis has achieved 98.15% accuracy in classifying tea grades [14], and portable systems have enabled rapid identification of high-pressure processing (HPP)-treated fish products [15]. The principles and application scenarios of NIR technology are shown in Figure 1.

### 1.3. Scope and Objectives of the Review

This review synthesizes insights from more than 134 peer-reviewed studies in recent years to explore the multifaceted applications of NIR spectroscopy in food analysis. It systematically examines: (1) the application of NIR in the food industry such as grains, meat, fruits, etc., for quality assessment and safety monitoring; (2) chemometric strategies to enhance spectral data interpretation, including preprocessing, feature extraction, and modeling; and (3) technological innovations driving practical implementation, such as portable devices and data fusion.

Although several review articles on the application of NIR in food analysis were published recently [13,16,17], most reviews primarily focused on summarizing conventional applications, introducing methods, and listing case studies, while lacking in-depth critical evaluation of inherent limitations, systematic comparison, and discussion of the relationship between NIR and traditional analytical methods (e.g., HPLC/ELISA). This review explicitly compares the differences and relationships between NIR and traditional validation methods, critically analyzes the advantages and applicable boundaries of different chemometric methods, and proposes targeted and hierarchical future development directions addressing actual industrial bottlenecks, rather than general outlook descriptions.

Moreover, by addressing the current challenges of model robustness and calibration transfer, as well as highlighting future directions for AI integration and multispectral fusion, this review aims to bridge the gap between academic research and industrial adoption, fostering the use of NIR spectroscopy as a standard tool for ensuring food quality and safety.

## 2. Fundamental Principles of NIR Spectroscopy in Food Analysis

### 2.1. Mechanism of NIR Spectral Generation

**Figure 2:**
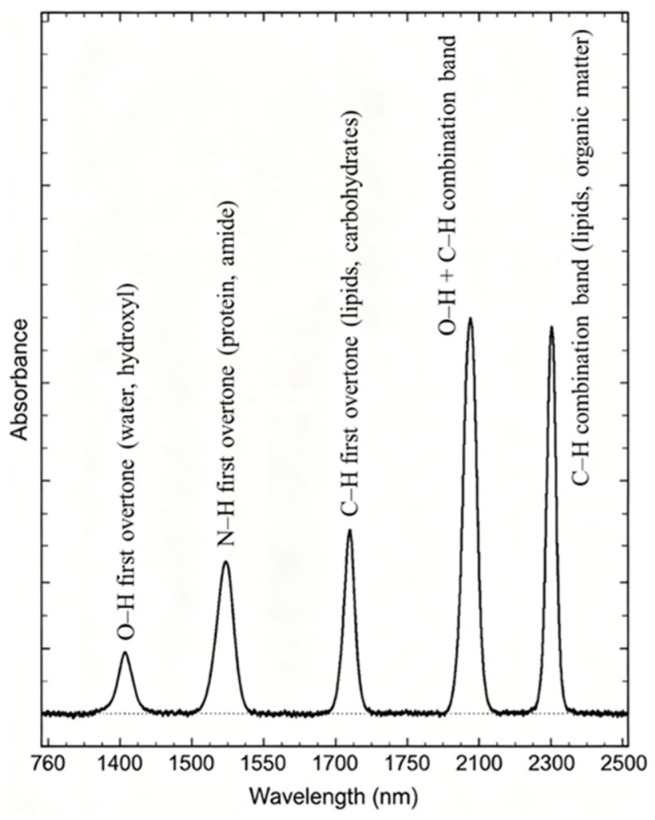
Schematic diagram of typical near-infrared absorption spectra of food samples.

The wavelength range of NIR spectroscopy is located between visible light and mid-infrared, and it is the core band for non-destructive food testing [18,19]. The schematic diagram of typical near-infrared absorption spectra of food samples is shown in Figure 2. NIR spectroscopy relies on the absorption of electromagnetic radiation in the wavelength range of 760–2500 nm, where molecular vibrations of hydrogen-containing functional groups produce overtone and combination bands [6,20]. These vibrations depend on the chemical composition and physical structure of food matrices, rendering NIR spectra sensitive to changes in nutrients, moisture, and key components [21,22]. For instance, the O–H stretching overtone near 1450 nm and C–H combination bands near 2300 nm are critical for quantifying water and fatty acids in meat [23], and N–H vibrations near 1550 nm support protein analysis in grains [24]. The diffuse reflectance or transmittance of NIR radiation through food samples is recorded, and chemometric models establish correlations between spectral features and target analytes [25].

However, these bands are broad, overlapping, and non-specific, which means that NIR cannot directly identify or quantify individual chemical compounds or specific functional groups. Instead, NIR spectra reflect the overall characteristics of macroscopic components and broad categories of substances, such as water, proteins, lipids, and carbohydrates in the food matrix. Therefore, NIR is generally not suitable for directly detecting trace substances, such as pesticides, toxins, or specific contaminants, unless supported by high-quality calibration models and chemometric algorithms.

### 2.2. Sampling and Measurement Techniques

#### 2.2.1. Reflectance and Transmittance Modes

Reflectance mode is suitable for solid or powdered samples, where NIR radiation is reflected from the sample surface [26]. Reflectance NIR has been applied to realize high-precision detection of aflatoxin B1 (AFB_1_) in maize [27] and evaluate the quality of matcha tea powder [28].

Transmittance mode is used for translucent samples, where radiation passes through the sample [29]. Transmittance NIR has been applied to measure soluble solids content in apples [30] and detect pesticide residues in lettuce leaves [31].

Tessaro et al. [26] compared the NIR transmission spectra of water-extracted coffee samples with the NIR reflection spectra of the corresponding ground samples to identify four geographically indicated coffees from southeastern Brazil: Cerrado Mineiro, Mogiana Paulista, Mantiqueira de Minas, and Matas de Minas. Using data-driven soft independent modeling of class analogy (DD-SIMCA), the results showed excellent classification performance. Although both acquisition modes showed satisfactory performance in calibration and validation, the reflection mode consistently outperformed the transmission mode in test set predictions, with accuracy ranging from 97% to 100%. The superior performance of the reflection mode models is due to their retention of chemical information features in the solid coffee matrix, including lipids and other compounds that are difficult to extract in water, whereas the water extracts are dominated by water absorption and exhibit greater intra-class variability.

#### 2.2.2. NIR Hyperspectral Imaging (HSI) Technique

As an advanced extension mode of NIR spectroscopy, HSI combines the spectral information and spatial distribution information of samples and has been increasingly applied to the analysis of complex food matrices. HSI can acquire complete near-infrared spectra at each pixel on the sample surface, enabling visualization of component distribution, localization of contaminated areas, and identification of quality differences. It is especially suitable for heterogeneous samples [32], locally contaminated samples [33], and products with complex appearances [34]. Combined with chemometric methods, NIR-HSI can achieve non-destructive, rapid, and highly accurate analysis of food safety and quality attributes.

#### 2.2.3. Sample Preparation for Solid, Liquid, and Powdered Foods

For solid food samples such as whole fruits, meat cuts, or intact grains, preparation often aims to ensure surface uniformity and consistent thickness. In the case of fruits like apples, any surface contaminants were gently wiped off, and samples were typically sliced to a uniform thickness to standardize the path length of NIR radiation [35,36]. When analyzing meat, removing external fat layers or connective tissues that could introduce variability is crucial. For instance, in a study on pork freshness using portable NIR spectrometers, samples were cut into 2–3 cm cubes to facilitate homogeneous measurement of muscle tissue properties [23].

Liquid samples, including milk, juices, and beverages, require careful handling to avoid measurement errors. A common approach is to use quartz cuvettes or flow cells. For tea liquid analysis, a 1–2 mm pathlength quartz cuvette was frequently employed to accurately measure the grades of tea via NIR transmittance [37,38]. When dealing with samples that may contain particulates, such as extracts of Sudanese karkade, filtration through a filter is advisable to prevent light scattering and ensure clear transmission of NIR radiation [39]. Additionally, maintaining a constant temperature during measurement is essential, as temperature can affect the refractive index and thus the spectral characteristics of the liquid sample.

Powdered foods like flour, tea powder, or powdered spices demand specific preparation steps. First, samples need to be homogenized to ensure uniform distribution of particles. Equipment can be used to blend powdered samples thoroughly. For example, when preparing instant corn powder mixtures, a high-speed grinder rotates the powder to achieve a homogeneous blend [40,41]. After homogenization, samples were packed into sample cups with consistent filling levels. In the analysis of cereal flours using NIR spectroscopy, the powder was gently filled into sample cups and leveled off to ensure consistent sample thickness, typically around 1–2 cm, to minimize light scattering variations during spectral acquisition [42].

### 2.3. Comparison Between NIR Spectroscopy and Conventional Analytical Methods

Traditional analytical techniques such as HPLC and ELISA are recognized as standard and authoritative methods for food quality and safety testing, offering high sensitivity, low limits of detection (LOD), excellent accuracy, and strong specificity. These methods are irreplaceable for regulatory inspection, confirmatory analysis, and quantitative detection of trace substances, including mycotoxins, pesticide residues, and illegal additives.

In contrast, NIR spectroscopy shows significant advantages in detection speed, non-destructive testing, minimal sample preparation, high throughput, and on-site applicability, making it suitable for real-time monitoring and large-scale screening in the food supply chain. However, compared to HPLC and ELISA, NIR has notable limitations in sensitivity, detection limits, absolute accuracy, and structural specificity. NIR signals originate from broad-band overtones and combination bands, which are easily influenced by matrix effects, sample conditions, and calibration models. Therefore, NIR cannot achieve the precision and reliability of standard chromatographic or immunological methods, especially for detecting trace contaminants.

In practical applications, NIR spectroscopy is complementary to traditional techniques rather than a replacement. NIR can serve as an efficient rapid screening tool for preliminary identification and classification, while positive or suspicious samples must be further verified through HPLC, ELISA, or chromatography-mass spectrometry coupled techniques to ensure food safety. Such combination strategies can significantly improve detection efficiency while meeting regulatory standards.

## 3. Applications of NIR Spectroscopy in Food Sectors

As a rapid, non-destructive and efficient analytical technique, NIR spectroscopy has been extensively applied in diverse sectors of the food industry, covering cereals and grains, meat and poultry products, fruits and vegetables, dairy and fermented products, as well as processed foods and beverages [43,44,45,46]. It fulfills multiple critical tasks in food quality and safety control, including quantitative analysis of nutritional components, detection of toxic contaminants, evaluation of freshness and texture properties, identification of adulteration, discrimination of varieties and geographical origins, and real-time monitoring of fermentation and processing procedures. This chapter systematically elaborates the practical applications of NIR spectroscopy in the above food categories, demonstrating its extensive adaptability and technical advantages in food analysis. Table 1 shows the specific applications and effects of NIR technology in the food field.

### 3.1. Cereals and Grains

As the most fundamental and widely consumed agricultural products, cereals and grains have become the primary field for NIR spectroscopy application and promotion. Benefiting from its high efficiency and non-destructive features, NIR is particularly suitable for large-scale quality screening of grain products.

#### 3.1.1. Quantitative Analysis of Nutrients

NIR spectroscopy has become a pivotal tool for the rapid quantification of key nutrients in cereals and grains, allowing non-destructive analysis of protein, lipid, moisture, and carbohydrate contents [59].

In maize kernel analysis, NIR coupled with partial least squares (PLS) regression has achieved high-precision prediction of protein content, with a coefficient of determination (R^2^) of 0.94 and root mean square error of prediction (RMSEP) of 0.87% [40]. Zhu et al. [60] conducted a study on the lipid content of oats based on the PLS regression spectral analysis model, with a standard error of prediction (SEP) of 0.3%. Pavas et al. [61] combined NIR spectroscopy with chemometrics to develop a rapid, non-destructive, and safe method for determining moisture and carbohydrates in pearl millet. The NIR models developed high-precision models for moisture (R^2^ = 0.995) and carbohydrates (R^2^ = 0.992).

These applications demonstrate that NIR has the potential to replace time-consuming wet chemistry methods in grain quality control and support real-time monitoring during storage and processing.

#### 3.1.2. Mycotoxin Detection

The detection of mycotoxins such as AFB_1_ in cereals is critical for food safety, and NIR spectroscopy has shown promise in rapid, sensitive screening. Deng et al. [62] developed a characteristic wavelength optimization strategy using particle swarm optimization combined with moving window (PSO-CMW) for AFB_1_ detection in maize, achieving an R^2^ of 0.97 and RMSEP of 3.5967 μg/kg. The study compared interval variable iterative space shrinkage approach (IVISSA), iterative retained information variable (IRIV), and PSO-CMW, demonstrating that PSO-CMW-SVM models offered the best generalization performance. Liu et al. [63] proposed a method for the quantitative detection of AFB_1_ in corn based on Fourier transform NIR (FT-NIR) spectroscopy. The results showed that the back propagation neural network (BPNN) model built using four characteristic wavelength variables optimized by the non-dominated sorting genetic algorithm (NSGA-II) had the best predictive performance, with the best NSGA-II-BPNN model having a prediction correlation coefficient (R_P_) of 0.9951 and a RMSEP of 1.5606 μg·kg^−1^.

For ochratoxin A detection in wheat, NIR hyperspectral imaging combined with deep learning has enabled spatial mapping of toxin contamination, with a classification accuracy of 92.3% [27]. These methods address the limitations of traditional HPLC by enabling rapid, non-destructive screening of large grain batches, crucial for preventing mycotoxin-contaminated products from entering the food chain.

Although NIR shows potential for the rapid screening of mycotoxins in cereals, it should be emphasized that NIR cannot replace authoritative confirmatory methods like HPLC. NIR provides an efficient, non-destructive tool for preliminary screening of large quantities of cereal samples, but it lacks the selectivity and sensitivity required for regulatory or confirmatory analysis. Positive results obtained through NIR must be further verified by standardized chromatographic methods to ensure food safety.

#### 3.1.3. Variety and Origin Discrimination

NIR spectroscopy has proven effective in discriminating cereal varieties and tracing geographical origins, leveraging spectral fingerprints of chemical profiles. In a study on red jujube varieties, a portable NIR spectrometer combined with fuzzy improved linear discriminant analysis (FiLDA) achieved 96.3% classification accuracy, outperforming traditional linear discriminant analysis [64]. For black goji berries (*Lycium ruthenicum*), NIR combined with competitive adaptive reweighted sampling (CARS) and PLS successfully discriminated samples from different regions based on anthocyanin content, with R^2^ values exceeding 0.90 [65].

In maize variety classification, fuzzy discriminant c-means (FDCM) clustering of NIR reflectance spectra achieved 97% accuracy, surpassing fuzzy c-means (FCM) and possibilistic c-means algorithms [66]. These applications demonstrate NIR’s utility in agricultural traceability and quality assurance, supporting sustainable supply chain management and consumer trust.

In summary, NIR can identify the varieties and origins of cereals and grains through stable spectral fingerprints.

### 3.2. Meat and Poultry Products

Meat and poultry products are susceptible to spoilage and adulteration during storage and processing, which urgently requires rapid and non-destructive detection methods. NIR spectroscopy provides reliable technical support for real-time quality control of meat products.

#### 3.2.1. Freshness Evaluation

NIR spectroscopy has emerged as a pivotal tool for non-destructive freshness assessment in meat and poultry, targeting key indicators like total volatile basic nitrogen (TVB-N) and total viable count (TVC). In a study on lamb freshness, Mia and Hashem [49] evaluated a method combining non-destructive NIR spectral measurements with machine learning to effectively classify fresh and frozen lamb. NIR spectra in the 700–1100 nm range were used, combined with eight optimized machine learning classifiers. Five-fold cross-validation further confirmed the robustness of the spectroscopy-based classification. Among them, XGBoost achieved the highest accuracy (91.2%), precision (90.1%), recall (92.6%), F1 score (91.3%), and ROC AUC (0.95), outperforming random forest and other models.

Ouyang et al. [23] employed a portable Vis-NIR system to detect cooking loss rate in frozen-thawed pork, achieving a prediction accuracy R_P_ of 0.8421 for thawed samples using CARS coupled with PLS. The study compared spectra from frozen and thawed pork, demonstrating that NIR can differentiate quality changes induced by freezing, with characteristic wavelengths at 980 nm (OH bond in water) and 1550–1650 nm (protein-related vibrations). For microbial quality monitoring, Kutsanedzie et al. [7] reviewed NIR applications in meat safety, highlighting its potential to quantify TVC in chicken using chemometric models like backpropagation artificial neural networks (BP-ANN), with R_P_ values exceeding 0.85. These methods replace traditional plate counting and chemical assays, enabling real-time freshness screening in slaughterhouses and distribution chains.

#### 3.2.2. Tenderness and Texture Prediction

NIR spectroscopy has shown promise in predicting meat texture attributes like tenderness and water-holding capacity (WHC), which are critical for consumer acceptability. Wei and Li [67] took shrimp as the research subject, collecting a total of 216 sets of NIR spectral data ranging from 680 to 2600 nm along with quality and texture indicators of the samples, and established a quantitative prediction model. The correlation coefficient (R) for cohesiveness in the validation set of the qualitative construction indicator was 0.868, and the R for elasticity in the validation set was 0.781. Vasconcelos et al. [68] evaluated the ability of NIR spectroscopy to estimate the WHC in the *Longissimus thoracis et lumborum* (LTL) muscle of Pizaaro pigs, and established a regression model using support vector machine (SVM). The WHC variables showed the estimation model (RMSE of 0.472%; R^2^ of 0.996; slope of 0.925 ± 0.006).

#### 3.2.3. Adulteration Detection

NIR spectroscopy combined with chemometrics has become a powerful tool for identifying meat adulteration, such as pork or poultry substitution in beef products. Pirhadi et al. [69] combined NIR spectrophotometer, principal component analysis (PCA), and linear discriminant analysis (LDA) to identify handmade sausage samples of beef, chicken, donkey, and horse meat. Through PCA, two principal components were extracted, covering 99% of the data variance, and were able to accurately distinguish sausage samples from different animal meats. Subsequently, the LDA model was able to confidently classify the sausage samples and achieved 100% accuracy in the classification of all test samples. Bai et al. [70] compared the NIR absorption bands of different meat varieties and developed a qualitative and semi-quantitative method for detecting various types of frozen meat adulteration based on NIR spectroscopy and chemometrics. The results showed that the PLS-DA model identifying all types of adulteration had the lowest accuracy (AUCP = 0.8265), while the PLS-DA models for lamb–chicken and beef–chicken had the highest accuracy (AUCP = 1). The AUCPs for other adulteration types were all above 0.92.

In summary, NIR can accurately predict freshness, tenderness, water-holding capacity and texture of meat and poultry products, and achieve high accuracy in identifying adulteration through classification models. However, its performance diminishes for frozen–thawed, high-fat, or highly heterogeneous samples.

### 3.3. Fruits and Vegetables

Fruits and vegetables feature complex appearances, diverse textures and short shelf life, bringing high requirements for postharvest quality detection. NIR spectroscopy achieves non-destructive internal quality evaluation without damaging the fruit appearance.

#### 3.3.1. Soluble Solids Content (SSC) and Acidity Measurement

NIR spectroscopy has become a standard tool for non-destructive quantification of SSC and acidity in fruits, enabling rapid quality assessment [71].

In a study on ‘Fuji’ apples, Guo et al. [72] compared shortwave and longwave NIR spectroscopy, achieving SSC prediction using independent component analysis-support vector machine (ICA-SVM) models. For tomatoes, model development for SSC using NIR at different storage temperatures achieved R_P_ =0.8988, by combining wavelet transform with PLS regression [73].

Su et al. [74] used visible/NIR (Vis/NIR) spectroscopy combined with chemometrics to quantitatively analyze SSC and total acidity (TA) of Ugni Blanc grapes non-destructively. Feature-based models outperformed full-spectrum models for TA, while full-spectrum models performed better for SSC. The $R_{P}^{2}$ values of the best models reached 0.957 and 0.913, and the residual predictive deviation (RPD) values were 4.798 and 3.397, respectively.

These applications demonstrate NIR’s utility in postharvest quality control for fresh produce.

#### 3.3.2. Pesticide Residue Screening

NIR spectroscopy has shown promise in rapid screening of pesticide residues and freshness indicators in vegetables. Lapcharoensuk et al. [75] combined NIR spectroscopy technology with machine learning algorithms to identify chlorpyrifos residues on bok choi. The most accurate model used SVM and principal component ANN (PC-ANN) along with raw spectral data, achieving 100% accuracy in classifying the chlorpyrifos residue content of the calibration set samples. Subsequently, the model was tested with 40 unknown datasets to verify its robustness, achieving a satisfactory F1 score of 100%. Zhang et al. [76] used Vis/NIR spectroscopy combined with PLS discriminant analysis (PLS-DA) and least squares SVM (LS-SVM) to develop two predictive models for detecting pesticide residues of abamectin, dichloroethane, and chloramphenicol at different concentration levels on cauliflower surfaces. Regression coefficients (RC), successive projections algorithm (SPA), and CARS were sequentially used for data dimensionality reduction. The results showed that RC-LS-SVM was the best discriminant model for detecting abamectin residue concentrations on cauliflower surfaces, with a prediction set discrimination accuracy of 98.33%. For detecting different concentrations of dichloroethane, SPA-LS-SVM had the best prediction accuracy of 95%. The CARS-PLS-DA-based model achieved an accuracy of 93.33% in identifying different concentrations of chloramphenicol on cauliflower surfaces.

NIR spectroscopy has shown potential for rapid preliminary screening of pesticide residues in vegetables and fruits. However, due to matrix interference, weak spectral signals of trace targets, and insufficient selectivity, NIR cannot serve as an independent confirmation method for regulatory purposes. It is suitable for on-site, high-throughput screening in the supply chain, while standard chromatographic methods such as LC remain indispensable for final quantitative confirmation.

#### 3.3.3. Postharvest Quality Monitoring

NIR spectroscopy facilitates high-accuracy discrimination of fruit varieties and geographical origins based on chemical profiles.

For the discrimination of varieties, Castillo-Girones et al. [77] explored methods to distinguish 17 strawberry varieties using Vis-NIR spectral imaging and ANNs, with a total of 3564 samples used. Three algorithms were evaluated, including SVM, XGBoost, and multilayer perceptron (MLP), among which the MLP model achieved the best classification performance, with an F1 score of 0.84. Lin et al. [78] proposed and validated a special design of a dual convolutional neural network (dCNN) for the problem of mango variety classification using NIR spectroscopy. During model testing, it outperformed all traditional methods, achieving a maximum accuracy of 89.34%.

For geographical origin tracing, goji berries from different regions were discriminated using NIR combined with LS-SVM, achieving more than 96.67%. classification accuracy [79]. Xia et al. [80] used NIR spectroscopy to analyze winter jujube samples from four different production areas, with a classification accuracy of 97.7% on the test set.

### 3.4. Dairy and Fermented Products

Dairy and fermented products involve complex biochemical changes during processing, making online monitoring difficult. NIR spectroscopy enables real-time tracking of key indicators during fermentation and processing.

#### 3.4.1. Milk Composition Analysis

NIR spectroscopy has been widely applied to quantify key components in dairy products, enabling rapid quality control in processing and storage [81].

In milk analysis, Suksangpanomrung et al. [82] conducted a comprehensive study on the effectiveness of NIR spectroscopy in assessing the quality of desiccated coconut throughout the entire ultra-high temperature (UHT) coconut milk production process. Using PLS models, the study successfully demonstrated the ability of NIR spectroscopy to accurately predict moisture, fat, protein, and pH levels in desiccated coconut. The PLS models showed calibration correlation coefficients (RC) exceeding 0.970, and the RPD ranged from 3.70 to 11.75, indicating excellent predictive capability. Khan et al. [83] explored the potential of a miniature NIR sensor based on a Fabry–Pérot interferometer (FPI) for in situ analysis to quantify macro components such as fat, protein, lactose, and total solids in raw milk. PLS regression was used to establish calibration models for the macro components of raw milk, and prediction performance was evaluated through statistical indicators. It was observed that it could quantify fat (RMSEP = 0.15%), protein (RMSEP = 0.15%), and total solids (RMSEP = 0.30%). However, the prediction accuracy for fat (RMSEP = 0.35%) and protein (RMSEP = 0.33%) was relatively low.

For yogurt quality assessment, Liu et al. [84] measured using NIR backscattering sensors at different fermentation temperatures and milk protein concentrations, and employed a mathematical model that correlates the light scattering signal with pH. This model has been successfully validated and can be used for both continuous and intermittent pH measurements, with SEP values all less than 0.09 pH units and coefficient of variation (CV) less than 1.78%. The proposed optical online non-destructive method enables online pH monitoring of the milk fermentation process, avoiding traditional manual pH measurements. Aktas et al. [85] compared the applicability of FT-NIR spectroscopy and basic rheological methods for online monitoring of lactic acid fermentation in *Portulaca oleracea*-fortified yogurt, while also evaluating the changes in yogurt properties during storage. By processing spectral data through principal component analysis and the Gompertz equation, kinetic critical points can be calculated. Applying the Gompertz equation to rheological data showed that FT-NIR spectroscopy could detect the fermentation process earlier, with the critical time on average about 18% earlier, thereby enabling better control over yogurt production.

These applications replace time-consuming wet chemistry methods, enabling online monitoring in dairy processing plants.

#### 3.4.2. Fermentation Stage Monitoring

NIR spectroscopy has emerged as a powerful tool for real-time monitoring of fermented food production, providing insights into key chemical transformations. In Zhenjiang aromatic vinegar production, NIR combined with PLS regression monitored total acid content and alcohol conversion during solid-state fermentation, achieving $R_{P}^{2}$ = 0.9760 for total acid prediction in the range of 900–1700 nm [86]. Zhang et al. [87] proposed a chemometrics framework based on NIR that explicitly incorporates fermentation round information to enhance the monitoring of physicochemical changes during the stacked fermentation of sauce-flavored baijiu. The GA-XGBoost model achieved a classification accuracy of 99.26% for fermentation round identification. The RFS-SVR (support vector regression) model accurately predicted acidity (R^2^ = 0.9674) and starch content (R^2^ = 0.9610).

In summary, NIR supports the online process control of the quality of dairy and fermented products, but it requires strict environmental stability.

### 3.5. Processed Foods and Beverages

Processed foods and beverages have diverse categories and complex formulations, which pose challenges to traditional detection methods. NIR spectroscopy achieves rapid grading, authenticity identification and quality assessment for such products.

#### 3.5.1. Tea and Coffee Quality Grading

NIR spectroscopy has become an efficient and widely used tool for quality control in processed foods, enabling rapid analysis of chemical profiles and authenticity [88,89].

In tea processing, Wu et al. [14] developed a fuzzy maximum uncertainty LDA (FMLDA) model for Chun Mee tea grade classification, achieving 98.15% accuracy by integrating NIR spectra with k-nearest neighbor (KNN) classification. The study identified key wavelengths at 7300–7100 cm^−1^ (OH/N-H bonds in tea polysaccharides and hydrogen-containing compounds) and demonstrated that FMLDA outperformed direct LDA (DLDA) and maximum uncertainty LDA (MLDA). Guo et al. [90] used NIR analytical methods for the simultaneous determination of active components and antioxidant capacity in green tea. The results showed that NIR combined with simulated annealing PLS (SA-PLS) and synergy interval PLS (Si-PLS) had a strong correlation with wet chemical methods in predicting epigallocatechin gallate ($R_{P}^{2}$ = 0.97), epigallocatechin ($R_{P}^{2}$ = 0.97), epicatechin gallate ($R_{P}^{2}$ = 0.96), epicatechin ($R_{P}^{2}$ = 0.91), catechin ($R_{P}^{2}$ = 0.98), caffeine ($R_{P}^{2}$ = 0.96), and theanine ($R_{P}^{2}$ = 0.93), as well as antioxidant capacity ($R_{P}^{2}$ = 0.80).

Setyaningsih et al. [91] evaluated NIR spectroscopy combined with machine learning algorithms, including support SVM, random forest (RF), and LDA, for the classification of pearl coffee beans. The results showed that SVM achieved 100% accuracy, while RF improved from 88.89% to 100% after feature selection, and LDA reached 97.92%. Kutsanedzie et al. [92] used FT-NIR spectroscopy combined with chemometric algorithms to predict the total fungal count (TFC) in cocoa bean solutions. The performance of the developed models showed 0.951 ≤ R_P_ ≤ 0.975; and 3.15 ≤ RPD ≤ 4.32. The prediction stability of the models increased in the order PLS < CARS-PLS < Ant colony optimization–PLS (ACO-PLS) < Si-PLS < synergy interval–genetic algorithm–PLS (Si-GA-PLS). FT-NIR spectroscopy combined with Si-GA-PLS can be used for in situ, non-invasive quantitative detection of TFC in cocoa beans for quality and safety monitoring.

#### 3.5.2. Quality Assessment of Nuts and Edible Oil Products

For nut products, Zareef et al. [93] applied benchtop NIR spectroscopy to predict antioxidant properties in walnuts, using Si-PLS combined with CARS. The model achieved R_P_ = 0.9616 for total phenolic content and R_P_ = 0.9657 for total flavonoid content. In processed grains, quantitative prediction of protein content in corn kernels via NIR achieved R = 0.93 with RMSEP = 0.3 g/kg, combining the PLS regression model with pretreatment methods [40]. Martínez-Peña et al. [94] evaluated the effects of orchard location and irrigation on the nutritional components of pistachios, and assessed the use of Vis/NIR hyperspectral imaging combined with machine learning techniques as a non-destructive prediction method. Pistachios from Moraleja de las Panaderas showed higher levels of nitrogen, phosphorus, protein, ash, and oleic acid, while samples from La Seca exhibited relatively higher levels of sodium and linoleic acid. Increased irrigation promoted the accumulation of various minerals and saturated fatty acids. Among the assessed algorithms, PLS regression performed the most stably and was able to accurately predict nitrogen (R^2^ = 0.75), zinc (R^2^ = 0.81), oleic acid (R^2^ = 0.91), linoleic acid (R^2^ = 0.87), ash (R^2^ = 0.81), carbohydrates (R^2^ = 0.87), and moisture (R^2^ = 0.84).

For edible oil products, Deng et al. [95] used a one-dimensional convolutional autoencoder (1D-CAE) to compress NIR spectral data to evaluate the antioxidant content in edible oils. Oil samples with different antioxidant concentrations were also characterized using FT-NIR spectroscopy. The experimental results showed that the best detection model established based on 1D-CAE compressed features achieved an average R^2^, RPD, and RMSE of 0.9953, 15.1664, and 1.2035 on the prediction set, respectively. Jiang et al. [96] developed a novel nano-modified colorimetric sensor combined with NIR spectroscopy for the detection of heavy metals in corn oil samples. The results showed that the sensor had high accuracy in detecting mercury and lead. The ACO-PLS model performed best for detecting low concentrations (10–100 ppb) of heavy metals. For the prediction of lead and mercury in corn oil containing interfering heavy metals (Mg^2+^, Zn^2+^, CO^2+^, Na^2+^, K^2+^), the $R_{P}^{2}$ values were 0.9793 and 0.9510, respectively, and the limits of detection (LOD) were 5 ppb and 7 ppb, respectively.

#### 3.5.3. Beverage Fermentation and Formulation Assessment

NIR spectroscopy has emerged as a pivotal tool for real-time monitoring in beverage fermentation and formulation.

Zareef et al. [97] used NIR spectroscopy to predict the concentrations of catechins, ferulic acid, gallic acid, L-epicatechin, astragaloside, and quercetin during the fermentation of Gongfu black tea, with RC and R_P_ ranging from 0.785 to 0.979 and 0.751 to 0.969, respectively. In Zhenjiang aromatic vinegar production, Fan et al. [86] used NIR to track total acid content and fermentation stages, achieving $R_{P}^{2}$ = 0.9760 with PLS regression. For kombucha fermentation, online NIR coupled with the sparrow search algorithm (SSA), whale optimization algorithm (WOA), and African vulture optimization algorithm (AVOA) predicted soluble sugar, total acid, and bacterial concentration, enabling dynamic adjustment of fermentation parameters [98].

In summary, NIR improves the quality control efficiency of processed foods and beverages, but it relies on standardized sampling.

## 4. Chemometric Methods for NIR Data Analysis

Raw NIR spectral data typically exhibit high dimensionality, information redundancy, and susceptibility to noise, light scattering, baseline drift, and complex matrix interferences, which hinder direct extraction of effective features linked to food attributes [99,100,101]. Chemometric methods form the core of NIR data analysis and cover the entire pipeline including data denoising, feature extraction, and model construction [102,103]. These strategies can effectively eliminate invalid interference, screen key spectral variables, establish reliable correlation models between spectra and target parameters, and significantly improve the accuracy, robustness, and practicality of NIR analysis [104,105]. This chapter systematically summarizes the main chemometric strategies applied in NIR food analysis, including spectral preprocessing, feature extraction, and variable selection, as well as common classification and quantitative modeling algorithms. Table 2 shows the specific chemometric methods for NIR data analysis.

### 4.1. Spectral Preprocessing Techniques

Spectral preprocessing is a critical step in NIR data analysis, aimed at eliminating irrelevant information (e.g., noise, light scattering, baseline drift) and enhancing the correlation between spectral features and target analytes. Common techniques include scatter correction, derivative transformations, and smoothing, which improve model robustness and accuracy across diverse food matrices.

#### 4.1.1. Scatter Correction Methods

Scatter correction techniques mitigate light scattering effects caused by differences in sample particle size, surface roughness, or packing density, which are particularly prominent in solid and powdered foods.

Standard normal variate (SNV) normalizes each spectrum by centering and scaling to remove multiplicative scatter effects, making it effective for heterogeneous samples. For example, SNV mitigated scattering effects, baseline shifts, and intensity differences related to sample geometry, enhancing the ability to distinguish between infested and sound asparagus spears [119].

Multiplicative scatter correction (MSC) corrects scatter by aligning spectra to a reference spectrum, reducing baseline shifts caused by physical properties. For example, using the MSC pretreatment method on NIR spectroscopy yielded the most accurate results in predicting the moisture and trypsin inhibitor content of soybean meal during the solid-state fermentation process of soybean meal [120].

#### 4.1.2. Derivative Transformations and Smoothing

Derivative transformations and smoothing techniques address baseline drift and high-frequency noise, enhancing spectral resolution for subtle chemical differences.

First- and second-order derivative transformations are often combined with S-G smoothing, to eliminate baseline offsets and emphasize spectral peaks associated with target analytes [121]. Techniques like S-G smoothing reduce random noise while preserving spectral features [122]. These preprocessing techniques are often combined to address multiple sources of interference, laying the foundation for robust chemometric modeling in food analysis.

### 4.2. Feature Extraction and Variable Selection

Feature extraction and variable selection are critical for simplifying high-dimensional NIR spectral data, reducing redundancy, and enhancing the performance of chemometric models. These techniques focus on identifying the most informative wavelengths or latent variables associated with target analytes, thereby improving model interpretability and robustness.

#### 4.2.1. Linear Feature Extraction Methods

Linear methods aim to transform or select variables while preserving linear relationships between spectra and analytes, making them suitable for datasets with low complexity.

PCA reduces dimensionality by projecting spectra onto orthogonal principal components (PCs) that capture the largest variance. It is widely used as a preprocessing step to eliminate noise and collinearity. For example, in apple variety classification, PCA compressed 1557-dimensional NIR spectra into a few PCs, which were then input to FDCM clustering, achieving 97% accuracy [66]. Similarly, PCA simplified spectral data for red jujube variety discrimination, enhancing the efficiency of FiLDA [64].

PLS extracts latent variables that maximize covariance between spectra and target variables, balancing dimensionality reduction and predictive power. For example, Arslan et al. [123] compared the application of NIR spectroscopy with PLS regression and various efficient variable selection algorithms, including Si-PLS, backward interval PLS (Bi-PLS), and genetic algorithm–PLS (GA-PLS), to predict the antioxidant activity in black wolfberry. Compared with full-spectrum PLS, the performance of the established models was significantly improved by applying Si-PLS, Bi-PLS, and GA-PLS. The R^2^ values for the calibration set and the prediction set ranged from 0.8479 to 0.9696 and from 0.8401 to 0.9638, respectively.

#### 4.2.2. Variable Selection Algorithms

Variable selection algorithms identify specific wavelengths or intervals that are most relevant to the target analyte, reducing model complexity and improving generalization.

Inspired by Darwinian evolution, CARS iteratively selects variables with high importance via Monte Carlo sampling and exponential decreasing functions. In the detection of SSC in sea buckthorn juice, CARS-PLS outperformed other methods, achieving an RMSEP of 1.42% and an RPD of 2.67 [124]. For walnut antioxidant properties, CARS combined with synergy interval PLS (Si-CARS-PLS) refined key wavelengths, resulting in R_P_ = 0.9616 for total phenolic content [93].

Interval selection methods partition spectra into intervals and select optimal combinations. Si-PLS was used to identify spectral intervals for total flavonoid content prediction, combining intervals at 4580–4860, 5720–6010, and 6290–6580 cm^−1^ to improve model accuracy [79]. Interval combination population analysis (ICPA) further enhanced variable selection by considering overlapping intervals, which was critical for the evaluation of matcha particle size and the ratio of tea polyphenols to free amino acids [125].

#### 4.2.3. Hybrid and Intelligent Optimization Methods

Hybrid methods integrate multiple algorithms to leverage their complementary strengths, often combining interval selection with evolutionary or fuzzy logic-based optimization.

PSO-CMW method uses PSO to optimize the position of moving windows, selecting continuous intervals with high predictive power. In AFB_1_ detection in maize, PSO-CMW identified 23 key wavelengths, enabling an SVM model to achieve $R_{P}^{2}$ = 0.9707 and RMSEP = 3.5967 μg/kg [62].

Fuzzy algorithms handle uncertainty in spectral data by assigning membership degrees to variables. For example, FMLDA extracted discriminant features from Chun Mee tea spectra, outperforming linear methods with a classification accuracy of 98.15% by capturing subtle spectral differences between grades [14].

The integrated interval variable selection method combines interval selection with variable selection to yield robust models for complex matrices. In matcha analysis, ICPA-CARS-PLS selected 28 variables for particle size prediction R_P_ = 0.9376 and 23 variables for polyphenol-to-amino acid ratio R_P_ = 0.9283, balancing model simplicity and accuracy [125].

These methods collectively address the challenges of high-dimensional NIR data, enabling precise and efficient analysis of food quality and safety attributes.

### 4.3. Classification and Quantitative Models

The choice of classification and quantitative models directly affects the accuracy and robustness of NIR spectral analysis. Depending on the linearity of spectral-data relationships, models are categorized into linear, nonlinear, and hybrid algorithms, each tailored to specific food analysis scenarios.

#### 4.3.1. Linear Models

Linear models are best suited for homogeneous samples and linear relationships (such as protein and moisture), and have the advantages of being simple, stable, and easy to interpret.

PLS regression is a staple in quantitative analysis, modeling the covariance between spectral data and analytes to handle multicollinearity. For example, PLS regression accurately predicted protein content in corn kernels with R_P_ = 0.93 [40]. In walnut antioxidant analysis, PLS combined with variable selection (Si-CARS) achieved R_P_ = 0.9616 for total phenolic content, demonstrating its efficacy in complex matrices [93].

LDA and its derivatives (DLDA, MLDA) are powerful for classification tasks. DLDA and MLDA were used for Chun Mee tea grade classification, achieving 96.3% accuracy, while their fuzzy extension (FMLDA) further improved accuracy to 98.15% by handling overlapping spectral features [14]. LDA also facilitated pork storage time discrimination, with correct classification rates exceeding 90% when combined with NIR spectra [126].

#### 4.3.2. Nonlinear Models

Nonlinear models outperform linear methods in complex matrices (such as meat, fruits, and soil-like matrices) because they can capture nonlinear spectral variations caused by scattering, moisture, and fat.

SVM achieves nonlinear classification or regression by mapping data to a high-dimensional feature space via kernel functions, making it effective for handling intricate spectral patterns. In the detection of aflatoxin B_1_ in maize, SVM combined with PSO-CMW variable selection outperformed linear models, yielding a prediction coefficient of determination $R_{P}^{2}$ = 0.9707 and RMSEP of 3.5967 μg/kg [62]. For matcha quality evaluation, it predicted the polyphenol-to-amino acid ratio with R_P_ = 0.9283 when combined with ICPA and CARS [125].

Artificial neural networks (ANN), especially BP-ANN and extreme learning machines (ELM), implement modeling of nonlinear relationships through layered networks of neurons, enabling them to perform adaptive learning from complex spectral data. BP-ANN was used to predict TVB-N in pork, achieving a R_P_ of 0.95, which outperformed linear PLS by capturing microbial spoilage-induced spectral changes [7]. In walnut antioxidant analysis, BP-ANN coupled with synergy interval (Si)-CARS variable selection predicted total flavonoid content with R_P_ = 0.9657, highlighting its ability to model phenolic compound-related spectral features [93]. ELM, a fast-learning variant of ANN, was employed in black tea fermentation monitoring, where it achieved R_P_ = 0.957 for catechin content, demonstrating superior efficiency compared to traditional BP-ANN [127].

Fuzzy neural networks integrate fuzzy logic’s ability to handle uncertainty with neural networks’ learning capability. It performs well in situations where classification boundaries are blurred (such as grade, origin, variety), and it can handle spectral overlap. For instance, FDCM clustering, which combines FCM with LDA, was used for apple variety classification. By assigning membership degrees to samples, FDCM achieved 97% accuracy, outperforming traditional FCM and possibilistic c-means (PCM) by better capturing subtle spectral differences between varieties [66]. Similarly, FiLDA enhanced red jujube variety discrimination, achieving 94.4% accuracy by accounting for vague boundaries in NIR spectra [64].

Moreover, although deep learning methods (e.g., CNN and ANN) show great potential in extracting subtle spectral features, they are highly prone to overfitting in NIR applications. Overfitted models perform excellently on the calibration set but their performance drops sharply in external validation and practical prediction, severely limiting their industrial applicability. Common reasons include limited sample size, the high dimensionality of full-spectrum data, excessive network layers, and over-learning of spectral noise rather than effective chemical information. To mitigate overfitting, effective strategies include reasonable training/validation sample division, spectral preprocessing to remove noise and baseline drift, wavelength variable selection to reduce redundant dimensions, network regularization, dropout configuration, and cross-validation optimization. In NIR analysis with deep learning, balancing model fitting capability and generalization ability remains a core challenge.

#### 4.3.3. Hybrid Algorithms

Hybrid models, which combine multiple algorithms, further boost performance by leveraging complementary strengths. For example, Adaboost combined with ULDA (Adaboost-ULDA) constructed a strong classifier, achieving 100% classification accuracy by an adaptive feature extraction process [35]. Additionally, Markov transition field (MTF) combined with CNN enhanced AFB_1_ detection in maize, with $R_{P}^{2}$ = 0.9955 by converting spectral sequences into spatial matrices for better feature extraction [27]. These hybrid approaches effectively handle the nonlinearity and complexity of food spectral data, improving robustness in real-world applications.

In summary, in practical food systems, nonlinear models and hybrid strategies are generally superior to linear models. However, linear models are more suitable for simple matrices. This is due to the obvious disadvantages of nonlinear and hybrid models, which include high model complexity, poor interpretability, and greater dependence on sample size and data quality.

## 5. Technological Challenges and Future Perspectives

### 5.1. Current Limitations

Despite the wide application of NIR spectroscopy in food analysis, several limitations remain, restricting its full industrial implementation. These challenges span technical constraints of instrumentation and spectral data, as well as methodological issues in model development and practical implementation. Furthermore, it should be emphasized that NIR spectroscopy cannot yet completely replace standard chromatographic or immunological methods, especially for trace contaminants, pesticide residues, mycotoxins, and certain specific chemical compounds. Its predictive performance is highly influenced by the complexity of the food matrix, the stability of the calibration model, and the quality of the reference dataset. Therefore, at present, NIR spectroscopy is more suitable as an efficient screening and preliminary assessment tool in the food supply chain, rather than as a definitive confirmatory technique.

#### 5.1.1. Technical Constraints of Instrumentation and Spectral Data

Although portable NIR spectrometers facilitate on-site analysis, they usually provide lower spectral resolution and stability than benchtop systems. For instance, portable Vis-NIR systems used for pork cooking loss rate detection showed slightly lower prediction accuracy for frozen samples R_P_ = 0.8154 compared to thawed samples R_P_ = 0.8421, partly due to reduced resolution in capturing subtle spectral variations induced by ice crystal formation [23]. Benchtop systems, though more accurate, are bulky and costly, limiting their use in real-time industrial lines.

Complex food matrices introduce significant spectral interferences, including light scattering from particle size heterogeneity and overlapping absorption bands. In matcha analysis, particle size variations caused scattering effects that required intensive preprocessing to mitigate, and even then, spectral overlap between polyphenols and amino acids challenged accurate ratio prediction [125]. Similarly, in aflatoxin B_1_ detection in maize, background noise from mold metabolites and matrix complexity necessitated advanced variable selection (e.g., PSO-CMW) to isolate relevant spectral features [62].

Spectra are highly sensitive to the physical states of samples. For example, frozen pork samples exhibited distinct spectral patterns at 980 nm compared to thawed samples, requiring separate calibration models to maintain accuracy [23]. This state dependence complicates model generalization across processing stages.

#### 5.1.2. Methodological and Practical Challenges

Calibration models often lack robustness across different batches, locations, or instruments. Long-term monitoring of apple soluble solids content (SSC) revealed that biological variability (e.g., ripeness, cultivar differences) caused model performance degradation over time, with R_P_ dropping from 0.92 to 0.85 within 6 months, highlighting the need for frequent recalibration [128]. Calibration transfer between benchtop and portable devices also remains problematic, as differences in spectral resolution lead to inconsistent feature extraction.

### 5.2. Emerging Trends

The application of NIR spectroscopy in food analysis is rapidly evolving, driven by advancements in instrumentation, algorithm development, and integration with other technologies. Emerging trends focus on enhancing accuracy, portability, and real-time monitoring capabilities, as highlighted in recent studies.

#### 5.2.1. Multimodal Data Fusion

Multimodal data fusion, integrating NIR spectroscopy with other sensing technologies, is gaining traction to capture comprehensive food quality information. This approach compensates for the limitations of single techniques by combining spectral, spatial, or odor data.

For example, in pork freshness monitoring, NIR spectroscopy was fused with electronic nose data to predict TVB-N, achieving a R_P_ of 0.9527, which outperformed single techniques (NIR alone: R_P_ = 0.8761; electronic nose alone: R_P_ = 0.649) [129]. Similarly, in moldy wheat analysis, NIR spectra combined with colorimetric sensor array data improved the quantification of colony counts, leveraging complementary chemical and visual information [130]. This trend extends to fermented foods. During kombucha fermentation, NIR was integrated with meta-heuristic algorithms to simultaneously monitor soluble sugar, total acids, and bacterial concentration, enabling dynamic process control [98].

#### 5.2.2. Advanced Intelligent Algorithms

The integration of deep learning, fuzzy logic, and hybrid optimization algorithms is revolutionizing NIR data analysis, enabling better handling of nonlinearity, high dimensionality, and complex food matrices.

Deep learning, such as CNN and transformer models are increasingly applied to extract intricate spectral features. For instance, MTF combined with CNN enhanced AFB_1_ detection in maize, converting spectral sequences into spatial matrices to capture long-range dependencies, resulting in $R_{P}^{2}$ = 0.9955 [27].

Fuzzy logic and hybrid models are also widely used in NIR spectroscopy data. Fuzzy algorithms, such as FMLDA and FDCM, improve classification accuracy by handling ambiguous spectral boundaries. FMLDA achieved 98.15% accuracy in Chun Mee tea grade classification, outperforming linear methods by accounting for overlapping spectral features [14]. Hybrid models like Adaboost-ULDA enhanced peanut origin traceability, achieving 95.06% accuracy by iteratively refining weak classifiers [131].

Meta-heuristic algorithms, such as PSO, CARS, and ICPA, are increasingly used to refine variable selection, reducing spectral redundancy and enhancing model interpretability. PSO-CMW has shown remarkable efficacy in selecting characteristic wavelengths for trace analyte detection. In the determination of aflatoxin B1 in maize, PSO-CMW identified 23 critical wavelengths, which, when input to an SVM model, yielded a $R_{P}^{2}$ of 0.9707 and RMSEP of 3.5967 μg/kg, outperforming other variable selection methods like IVISSA and IRIV [62]. CARS, inspired by Darwinian evolution, is widely applied to isolate informative variables in complex matrices. In the prediction of antioxidant properties (total phenolic content, total flavonoid content) in walnuts, CARS combined with Si-PLS (Si-CARS-PLS) selected 17–22 key wavelengths, achieving R_P_ values of 0.9616–0.9683 and RPD values of 2.728–3.807, which significantly improved model robustness compared to full-spectrum PLS [93]. ICPA, which considers overlapping spectral intervals to preserve continuous chemical information, has been integrated with CARS (ICPA-CARS) for matcha quality evaluation. This hybrid approach selected 23–28 variables for predicting particle size and polyphenol-to-amino acid ratio, resulting in R_P_ values of 0.9283–0.9376 and enhancing model efficiency by reducing redundant variables [125]. These meta-heuristic methods, by adaptively exploring the spectral space, address the “curse of dimensionality” in NIR data, enabling more accurate and stable predictions for diverse food attributes, from mycotoxins to antioxidants.

#### 5.2.3. Portable and Online Monitoring Systems

The demand for real-time, on-site food quality analysis has driven the development of portable NIR spectrometers and integrated online monitoring systems, enabling rapid decision-making throughout the food supply chain.

Portable NIR devices, characterized by their compact size, low cost, and ease of operation, are increasingly deployed for field and in-plant analysis. For example, in tea processing, portable NIR spectrometers combined with FiLDA discriminated red jujube varieties with 96.3% accuracy, facilitating rapid variety identification during harvesting and grading [64]. Similarly, portable systems monitored chlorophyll changes during Tencha processing, providing real-time feedback for optimizing tea leaf handling [132].

Online NIR monitoring systems, integrated into production lines, enable continuous quality assessment during manufacturing. For instance, in dough fermentation for Chinese steamed bread, NIR spectroscopy coupled with supervised learning algorithms monitored amylase activity and amino acid formation in real time, ensuring consistent product quality [133].

These advancements in portable and online systems are transforming food quality control from laboratory-based testing to real-time, in-process monitoring, reducing delays and enhancing efficiency in industries such as meat processing, beverage production, and cereal manufacturing. Future developments will focus on improving the stability of portable devices, expanding their application to complex matrices, and integrating them with Internet of Things (IoT) platforms for smart factory automation.

In short, the continuous integration of multimodal data fusion, intelligent algorithms, and portable/online devices has greatly elevated the detection accuracy, applicability, and real-time performance of NIR spectroscopy. These advancements are gradually overcoming traditional bottlenecks and laying a solid foundation for the wider industrial deployment of NIR technology in food quality and safety control.

### 5.3. Industrial Translation and Standardization

The transition of NIR spectroscopy from laboratory research to industrial applications, coupled with the establishment of standardized protocols, is critical for ensuring consistency, reliability, and widespread adoption in food quality control. This section addresses the progress and challenges in industrial translation and the development of standardized practices.

#### 5.3.1. Industrial Adoption and Technological Adaptation

The industrial translation of NIR spectroscopy hinges on the adaptation of technology to real-world production environments, emphasizing portability, robustness, and integration with existing workflows. Portable NIR devices have emerged as key tools for on-site quality checks, enabling rapid decisions in settings ranging from farms to processing plants. For instance, portable NIR spectrometers have been deployed for real-time detection of cocaine and its common harmful adulterants, thereby reducing reliance on time-consuming chromatographic analyses [134]. Similarly, portable systems have been used to monitor chlorophyll changes during Tencha processing, providing instant feedback to optimize tea leaf handling and ensure consistent product quality [132].

Online NIR monitoring systems have been integrated into production lines to enable continuous quality control. In kombucha fermentation, online Vis-NIR spectroscopy combined with meta-heuristic algorithms tracks soluble sugar, total acids, and bacterial concentration in real time, allowing dynamic adjustments to fermentation parameters and reducing production losses [98]. Similarly, NIR-based monitoring of dough fermentation in Chinese steamed bread processing has streamlined production by providing real-time data on amylase activity and amino acid formation, ensuring uniform product quality [133].

Despite these advances, industrial adoption faces challenges such as device stability under harsh production conditions (e.g., temperature fluctuations, dust), high initial investment costs, and the need for operator training to interpret spectral data. Recent efforts to develop low-cost, ruggedized NIR sensors and user-friendly software interfaces are addressing these barriers, facilitating broader implementation across small and medium-sized enterprises. In addition, it is crucial to establish a standardized workflow for the uniform implementation of industrial NIR. This workflow should include standardized sample preprocessing, consistent spectral acquisition parameters, a unified preprocessing procedure, rigorous model calibration and external validation, regular model updates, and long-term maintenance of calibration transfer. Uniform operating procedures can greatly reduce data variability across laboratories and production lines, enhancing the repeatability and practical applicability of NIR models.

#### 5.3.2. Standardization of Protocols and Calibration

Standardization is essential to ensure reproducibility of NIR-based results across laboratories, instruments, and production sites. Key areas of standardization include spectral acquisition protocols, calibration transfer methods, and data sharing frameworks.

Spectral acquisition protocols have been standardized for specific commodities to minimize variability. For example, studies on apple quality assessment have established guidelines for sample preparation (e.g., peel thickness adjustment) and spectral collection parameters to ensure consistency. Unified procedures for sample pretreatment, spectral collection, and model validation can effectively reduce deviations caused by different operators, instruments, and environmental conditions.

Calibration transfer remains a core issue in standardized application. Developing universal calibration models and efficient transfer algorithms will enable consistent prediction performance among different NIR devices, laying the foundation for cross-instrument and cross-region detection. In addition, establishing open spectral databases and shared model platforms will further promote the standardization and large-scale application of NIR technology in the food industry. However, standardized blanks still limit large-scale industrial promotion. At present, the international unified standards for NIR detection methods in the field of food safety are not yet complete. There is a lack of consistent regulations regarding instrument performance parameters, spectral acquisition specifications, model evaluation standards, and calibration transfer protocols. In addition, NIR has not yet been officially recognized as an official screening method in many food regulatory systems, resulting in limited acceptance in official testing and market supervision.

## 6. Conclusions

NIR spectroscopy combined with advanced chemometric algorithms has become a transformative technique for food quality and safety analysis, providing non-destructive, rapid, and multi-parameter detection across diverse food matrices. This review highlights its extensive applications in meat, fruits, vegetables, dairy, fermented products, and processed foods, demonstrating its versatility in quantifying chemical compositions, assessing freshness, predicting texture attributes, and detecting adulteration or contaminants.

Key methodological advancements, including spectral preprocessing, feature extraction, and modeling techniques, have significantly enhanced prediction accuracy and robustness. Nonlinear models and hybrid approaches have proven particularly effective in handling complex spectral data from heterogeneous samples, such as matcha particle size analysis and walnut antioxidant property prediction.

Despite current limitations such as instrument limitations, spectral interference, and challenges in model generalization, emerging trends such as multimodal data fusion, advanced intelligent algorithms, and portable/online monitoring systems are addressing these gaps. These innovations facilitate real-time quality control in industrial settings, from kombucha fermentation monitoring to on-site pork freshness assessments.

Industrial translation and standardization efforts, including the development of ruggedized portable devices and standardized protocols for spectral acquisition and calibration transfer, are critical for broader adoption. With continuous development, the integration of NIR spectroscopy with IoT and smart factory systems will revolutionize food supply chains, strengthen traceability, and better protect consumer health.

In summary, NIR spectroscopy, empowered by advancements in chemometrics and technology, is poised to become an indispensable tool in modern food science, bridging laboratory research and industrial applications to meet the growing demands for efficient, accurate, and sustainable quality control.

## Figures and Tables

**Table 1:** Typical applications and analytical performance of NIR spectroscopy in different food categories.

Subjects	Bands (nm)	Targets	Results	References
Teff	1100–2500	Detecting adulteration	The quantitative limits are 11% (ramie), 0.68% (carbohydrates), 0.20% (lipids), 0.08% (ash), and 0.36% (protein).	[47]
Wheat	570–1100	Determining cadmium concentration	PLS regression achieved a RMSE of 0.082 mg/ kg and a R_P_ of 0.952.	[48]
Lamb meat	700–1100	Distinguishing fresh and frozen lamb	XGBoost achieved the highest accuracy (91.2%), precision (90.1%), recall (92.6%), F1 score (91.3%), and ROC AUC (0.95).	[49]
Chicken meat	1000–2500	Detecting adulteration	The raw data model achieved a R_P_ of 0.91, a RMSEP of 0.25 mg/g, a performance-to-deviation ratio of 2.28, and a REP of 4.63%.	[50]
Blueberry	400–1000	Identification	Full-spectrum modeling achieved the highest accuracy (97.4%) in Vis/NIR data, while LDA performs best (94.5%) in NIR data.	[51]
Cherry tomatoes and strawberries	900–1700	Testing pesticide content	In cherry tomato and strawberry samples, the ranges of the coefficient of determination, RMSECV, and RPDCV are 0.83 to 0.93, 0.61 to 0.86; 0.01 to 0.03, 0.00 to 0.10; 2.45 to 3.80, 1.61 to 2.67, respectively.	[52]
Milk	410–940	Detecting adulteration	The classification model achieved an accuracy of >= 99%, with an inference time of less than 0.15 milliseconds.	[53]
Beer	1100–2500	Evaluating quality	The model demonstrated good predictive performance for actual extracts (RPRE = 0.97), specific gravity (RPRE = 0.95), color (RPRE = 0.96), pH value (RPRE = 0.92), and total IAA content (RPRE = 0.88), with an RPD exceeding 2.5.	[54]
Matcha	900–1700	Identifying the origin	The NIR-KNN model showed a 98.7% accuracy rate in origin identification.	[55]
Coffee	830–2500	Predicting moisture content	The PCR chemometric-based model allowed for an accurate prediction of moisture content (R^2^ >98% and RMSE < 3.4%).	[56]
Almond	908–1676	Detecting adulteration	Using portable and benchtop spectrometers to collect NIR spectra also demonstrated superior performance in predicting apricot kernels with PLSR, with a R_P_ > 0.96 and a prediction standard error of 3.98%.	[57]
Edible oil	1100–2498	Evaluating the oxidative capacity of cooking oil during the frying process	The optimized NIR-SELECT-OLS model demonstrated strong predictive performance across various oils (R^2^ > 0.90; explained variance > 85%).	[58]

**Table 2:** Common chemometric methods and their application performance in NIR spectral data analysis for foods.

Subjects	Bands (nm)	Chemometric Methods	Results	References
Honey	680–2600	LDA, SVM, PLS-DA	In the case of adulterated brown syrup, PLS achieved an R^2^ of 0.997 and an RMSECV of only 1.6593.	[106]
Milk	1000–2500	OPLS-XGBoost	The model showed high predictive performance, with RMSE, NRMSE, and CV-R^2^ for urea being 0.01, 0.02, and 0.97, respectively; for ammonium sulfate, 0.01, 0.02, and 0.96, respectively; for sugar, 0.07, 0.13, and 0.95, respectively; and for hydrogen peroxide, 0.01, 0.03, and 0.94, respectively.	[107]
Meatball	885–1679	PCA, PLS-DA, LDA, SVM, KNN, ANN, CNN, AlexNET, ResNET	Regardless of the features and algorithms used, halal meatball samples can be predicted and distinguished from non-halal meatball samples, with an overall prediction accuracy of up to 100%.	[108]
Cassava root and wheat-flour	400–2498 and 400–2496	CW-DFF-MBR	The proposed model provided stable predictive performance and consistent model interpretation on both datasets.	[109]
Chocolate bar	950–1600	PLS-DA	The model effectively distinguished between fresh and spoiled hazelnut chocolate bars (100% accuracy at the chocolate bar level).	[110]
Multiple legume species	1100–2498	MPLS and 1D CNN	The 1D CNN model outperformed MPLS, achieving R^2^ = 0.883 and RPD = 2.932, compared to MPLS with R^2^ = 0.814 and RPD = 2.320.	[111]
Chewable gel	1000–2500	PLS	The model demonstrated excellent predictive performance (R^2^ = 0.977, RPD = 6.9, RSEP = 4.1%) and high precision (RSD < 2.1%).	[112]
Minced meat	400–1100	MLP	The model achieved${\;R}_{cv}^{2}$ and $R_{P}^{2}\;$ of 0.879 and 0.916, respectively, in sample authenticity prediction. In quality grading, its accuracy was 92.4%, with precision, recall, and F1 score all reaching 96.2%.	[113]
Extra virgin olive oil	300–4000	PLS-DA	The model achieved 100% classification accuracy and has good predictive performance (R^2^ = 0.97, RMSEC = 5.90, RMSECV = 5.29).	[114]
Pacific sardine and mackerel	/	PLS, ANN and KNN	The model achieved a classification accuracy of ≥80% when identifying the youngest or oldest fish.	[115]
Cider	1100–2300	PLS	The model achieved R^2^ values of 0.81 to 0.86 in predicting the total main sugars, fructose, and glucose content.	[116]
Olive oil	190–1100	PLS and SVM	The PLS model achieved the highest predictive accuracy, with an R^2^ value exceeding 0.9970 and an RMSE value below 2%.	[117]
Pistachio	400–1000	PLS-DA and MLP-ANN	PLS-DA achieved high discrimination among the four sources, with an overall test set accuracy of over 98% for bulk and powdered samples, while the accuracy for individual kernels was slightly lower (86%). The MLP-ANN model confirmed the high predictive potential, with similar accuracy (>90%), especially for ground samples, where accuracy could reach 100%.	[118]

## Data Availability

No new data were created or analyzed in this study.

## Abbreviations

The following abbreviations are used in this manuscript:

1D-CAE	One-dimensional convolutional autoencoder
AFB1	Aflatoxin B1
AVOA	African vulture optimization algorithm
BP-ANN	Backpropagation artificial neural networks
BPNN	Back propagation neural network
CARS	Competitive adaptive reweighted sampling
CV	Coefficient of variation
dCNN	Dual convolutional neural network
DD-SIMCA	Data-driven soft independent modeling of class analogy
DLDA	Direct LDA
ELISA	Enzyme-linked immunosorbent assay
FDCM	Fuzzy discriminant c-means
FCM	Fuzzy c-means
FiLDA	Fuzzy improved linear discriminant analysis
FMLDA	Fuzzy maximum uncertainty LDA
FT-NIR	Fourier transform NIR
HPLC	High-performance liquid chromatography
ICA	Independent component analysis
ICPA	Interval combination population analysis
IRIV	Iterative retained information variable
IVISSA	Interval variable iterative space shrinkage approach
KNN	K-nearest neighbor
LDA	Linear discriminant analysis
LOD	Limits of detection
LS-SVM	Least squares SVM
LTL	Longissimus thoracis et lumborum
MLP	Multilayer perceptron
MLDA	Maximum uncertainty LDA
MSC	Multiplicative scatter correction
NIR	Near-infrared
NSGA-II	Non-dominated sorting genetic algorithm
PCA	Principal component analysis
PC-ANN	Principal component ANN
PLS	Partial least squares
PLS-DA	PLS discriminant analysis
PSO-CMW	Particle swarm optimization combined with moving window
R	Correlation coefficient
R^2^	Coefficient of determination
RC	Regression coefficients
RF	Random forest
RMSEP	Root mean square error of prediction
RP	Prediction correlation coefficient
RPD	Residual predictive deviation
SA-PLS	Simulated annealing PLS
S-G	Savitzky–Golay
SEP	Standard error of prediction
SNV	Standard normal variate
SPA	Successive projections algorithm
SSA	Sparrow search algorithm
SSC	Soluble solids content
Si-PLS	Synergy interval PLS
SVM	Support vector machine
TA	Total acidity
TVB-N	Total volatile basic nitrogen
TVC	Total viable count
UHT	Ultra-high temperature
WHC	Water-holding capacity
WOA	Whale optimization algorithm
